# The effect of active breaks on cognitive performance and classroom behaviour: the I-move study

**DOI:** 10.1093/eurpub/ckac129.518

**Published:** 2022-10-25

**Authors:** A Masini, M Ricci, S Marini, A Ceciliani, G Barone, D Gori, L Bragonzoni, A Sansavini, A Tessari, L Dallolio

**Affiliations:** 1 Department of Biomedical and Neuromotor Sciences, Alma Mater Studiorum - University of Bologna, Bologna, Italy; 2 Department of Life Quality Studies, Alma Mater Studiorum - University of Bologna, Rimini, Italy; 3 Department of Psychology “Renzo Canestrari”, Alma Mater Studiorum - University of Bologna, Bologna, Italy

## Abstract

**Background:**

Active Breaks (ABs) intervention involves short bouts of moderate to vigorous physical activity (MVPA) conducted during or between curricular lessons by the appropriately trained teachers. The aim of the Imola Active Breaks Study (I-MOVE study) was to evaluate the effect of an ABs intervention on cognitive function and classroom behaviour in primary school children.

**Methods:**

The study was quasi-experimental, and it involved two groups attending a primary school in Imola (Bologna, Italy). The Active Breaks group (ABsG) performed the I-MOVE protocol consisting in 10 minutes of ABs divided in warm up, tone-up with high intensity interval training and cool-down. This is repeated three times a day for one year and half. The control group (CG) continued with regular lessons. The baseline assessment was conducted in October 2019 and the follow-up in May 2021. Cognitive performance was assessed using working memory test and classroom behaviour was monitored using an “ad hoc questionnaire”.

**Results:**

Working memory performance increased significantly more in the ABsG (change: 1.30±1.17) than in CG (0.96±1.20), p < 0.05. Almost the entire sample of the children wanted to continue with this intervention in the next following year. Children reported improvements in their school-life quality, including feeling better in class (75.40%) and in school (82.50%) when using ABs. Improvements were also reported in children time-on-task behaviours: 52.90% said they work easily in class, 52.90% that they could listen more clearly, 58.80% reported they can stay seated easily, and 59.60% that they learned better and were more focused after ABs.

**Conclusions:**

In conclusion the program has proven to be very effective on the children's cognitive improvement and classroom behaviour. Since the ABs intervention demonstrates these positive effects, its implementation in schools can have a beneficial, sustainable and long-term impact on childhood health.

**Key messages:**

• ABs intervention represents a cost-effective strategy to be implemented in the school settings regardless of the age and sex differences, to make the school a more dynamic environment.

• Despite the pandemic difficulties, the ABs intervention proved to be sustainable, and to have a positive effect on classroom behaviour by improving children’s concentration and attention in class.

